# Potentials of Biochars Derived from Bamboo Leaf Biomass as Energy Sources: Effect of Temperature and Time of Heating

**DOI:** 10.1155/2019/3526145

**Published:** 2019-12-14

**Authors:** Bidayatul Armynah, Dahlang Tahir, Monalisa Tandilayuk, Zuryati Djafar, Wahyu H. Piarah

**Affiliations:** ^1^Department of Mechanical Engineering, Hasanuddin University, Bontomarannu Gowa 92171, Indonesia; ^2^Department of Physics, Hasanuddin University, Tamalanrea Makassar 90245, Indonesia

## Abstract

Biochars from bamboo leaves as a potential energy resource were synthesized by annealing in the oxygen-free environment. Samples were characterized using proximate analysis, Fourier-transform infrared (FTIR), X-ray diffraction (XRD), scanning electron microscopy (SEM), and energy-dispersive spectroscopy (EDS). Heating temperatures are 250°C, 300°C, and 350°C and for each temperature, the time was varied between 30, 60, and 90 minutes. The heating time for 30 minutes results in FC 30.777% and calorific value 15 MJ/Kg at temperature 250°C and decreased to 4.004% and 6 MJ/Kg at temperature 350°C, respectively. EDS shows the time of heating is an important parameter which shows the carbon and nitrogen contents were decreasing with the increase in the heating time, and silicon and oxygen contents were increasing with increase in the heating time. XRD shows broad (002) reflections between 20° and 30°, which indicated disordered carbon with small domains of coherent and parallel stacking of the graphene sheets, which is consistent with surface morphology of the SEM image. The experimental results indicated that heating at 300°C for 30 minutes is an effective and efficient parameter for fabrication of low-cost carbon from bamboo leaves which is a source of useful energy.

## 1. Introduction

Biomass is abundant in nature and contains varying organic compounds, which is a promising potential sustainable source of energy alternatives [1, 2]. The source of biomass can range from agricultural, industrial, and municipal products [2–4]. Biomass is becoming increasingly popular worldwide because of biodegradability and lower greenhouse gas emission ensuring reduced environmental pollution. Biomass is not only important as a source of energy but also as a source of different valuable fuels and chemicals [3, 4]. Biomass consists of different celluloses, hemicelluloses, lignin, and some other carbohydrates [5]. Bamboo leaves are one of the agriculture products which are still investigated for use as a source of energy [6–8]. Bamboo is a woody-stemmed grass which are distributed across approximately in 31.5 million ha [9] of land, concentrated in China and India [10]. In China, it occupied approximately 4.84–5.71 million ha, and in India, it occupies approximately about 5.48 million ha with about 80% found in the Asia-Pacific region [11]. Bamboo is cultivated in Asian countries. Bamboos are composed of hemicellulose, cellulose, and lignin that have great potential as a source of energy [6, 8]. To improve the quality of biomass, many kinds of processing application have been used by several researchers by using the following processes: torrefaction [8, 12, 13], pyrolysis [14–16], gasification [17], and sintering or bed sintering [18] to produce biochar or charcoal or black carbon or carbon nanotube [2–8, 12–18].

Torrefaction used the low-temperature thermal method between 200°C and 400°C under the atmospheric pressure and absence of oxygen. Biochar product can be analyzed by the proximate method to determine the fixed carbon, volatile, ash, and moisture under different heating conditions. Some study has been performed by using proximate analysis at high or low temperatures [4], predicting high heating value [13, 17] and heating behavior [8, 19] for different kinds of biomass, microalga [8, 20], canola, oat, palm, and Jatropha waste, and natural forest [1, 6–8, 14, 18, 21]. Some studies have reported a correlation between the result of the proximate analysis for predicting the elemental composition of carbon, hydrogen, and oxygen of biochar from biomass. An elemental composition which defines the energy content is an important property of biomass. Also, there is an increasing awareness of the importance of understanding the chemical bonding of biochar to optimize its role in energy [3, 14, 21]. The effect of temperature and time during heating on the chemical bonding, elemental composition, calorific value, and interactions between minerals inside the biochar is still far from being understood, and in-depth knowledge is needed for an effective evaluation of the use of biochar as a source of energy. Recent studies [4, 6, 16, 22, 23] suggest that the types of bonding mechanism during the interactions (e.g., absorption or transmission) and calorific value as source of energy depend on the following factors: (i) feedstock composition, in particular, the total percentage elemental composition; (ii) heating process conditions; (iii) biochar structural properties and crystallite size; and (iv) local environmental conditions. There is an increasing awareness of the importance of understanding the elemental composition (carbon, hydrogen, and oxygen), chemical bonding, and crystallite size of biochar to optimize its role as a source of energy.

In this study, the bamboo leaves as a source of carbon from Hasanuddin University campus was used to carry out the experiments as a function of heating temperature and time. We used proximate analysis to determine volatile matter (VM), ash, fixed carbon (FC), and moisture content (MC). By using three existing predicting formulas based on proximate analysis data as input parameter, we predict carbon, hydrogen, and oxygen content. We compared our result from EDS for carbon and oxygen content with the result of the predictive formula. The chemical bonding and structural properties of biochar are analyzed by using Fourier-transform infrared (FTIR) spectroscopy and X-ray diffraction spectroscopy (XRD), respectively.

## 2. Materials and Methods

In this study, we used the proximate analysis to determine ash, moisture, volatile matter, and fixed carbon from dried bamboo leaves around Hasanuddin University campus, Tamalanrea, Makassar, Indonesia. Dried bamboo leaves were cleaned by using distilled water to remove dust and kept in an open box at room temperature for 5 days. Bamboo leaves were cut into small pieces, grinded, and sieved to 500 *μ*m to guarantee homogeneity and then heated at 250°C, 300°C, and 350°C for 30, 60, and 90 minutes in an oxygen-free environment, respectively.

We have used proximate analysis based on ASTM standards *E* 871, *E* 1755, and *E* 872, for moisture, ash, and volatile matter, respectively. Fixed carbon content was calculated by %FC = 100—(%ash + %VM), where %FC, %ash, and %VM are the mean of the mass percentage of fixed carbon, ash, and volatile matter of the sample, respectively [24–26].

Infrared spectroscopy was carried out using an IRPrestige-21 FT-IR spectrometer (Shimadzu Corp) equipped with a bright ceramic light source, a KBr beamsplitter, and a deuterated triglycine sulfate doped with an L-alanine (DLATGS) detector. The measurements of the sample were collected over the range of 4000–600 cm^−1^ and 16 coadded scans. All samples were ground into powders prior to the spectral acquisition. All spectra were in transmittance units.

The X-ray diffraction (XRD) pattern was collected on an X-ray diffraction (XRD) spectroscopy (Shimadzu 7000) with Cu K*α* radiation (*λ* = 1.5405 Å) over the angular range of 15° ≤ 2*θ* ≤ 80°, operating at 30 kV and 10 mA. It was performed to examine the structural properties of the samples.

Morphological and chemical characterization (composition of carbon, oxygen, nitrogen, and another element in the samples) of particles has been performed by a scanning electron microscopy energy-dispersive X-ray spectrometry (SEM-EDS) (JEOL JSM-IT- 300) with an acceleration voltage of 10 kV and a beam current of 7.475 nA, and the lowest vacuum is 50 pa.

## 3. Results and Discussion

Table 1 shows volatile matter, moisture, ash, and fixed carbon of biomass from bamboo leaves with heating temperatures and time varied at 250˚C, 300˚C, 350˚C and 30, 60, and 90 minutes, respectively. For composition of ash at the temperatures 300°C and 350°C shows increases with increasing heating time due to hemicellulose, cellulose, and lignin were released gases in the form of CO_2_ and CH_4_ [6, 16, 23]. The temperature of 350°C for 90 minutes showed a discoloration in the samples from black to gray which indicates the ash content is high which has contributed to reduction of the fixed carbon composition. The highest composition of the volatile substance is 38.08% for temperature and time of 250°C and 90 minutes, respectively. Increase in moisture from 60 minutes to 90 minutes is typical probably because the time of heating is too long which affected the amount of water lost during the heating processes. Some studies reported the fixed carbon increase with increasing heating temperature, which is not observed in this study because the mass density of bamboo leaves is lower than that of another source of biomass [4, 6–8]. The most abundant ash elements in bamboo leaf biomass from X-ray fluorescence (XRF) spectroscopy in this study are Si, Ca, K, and P with compositions of 58%, 22%, 15%, and 5%, respectively.

Heating at 250°C for 90 minutes shows the highest volatile matter which may due to faster cooling during the experiment, and then the crucible is taken out from the furnace several minutes after the heating processes. The effect of faster cooling may increase the yield of volatile matter at 250°C for 90 minutes. The heating time also affects the calories produced, and the calories decreased with increasing heating time. Time at which highest calorie is produced is 30 minutes for every temperature considered in this study, as shown in Table 2. We compared the calorific value determined by using the bomb calorimeter in this study with the predictive model developed by Majumder et al. [27] in the form of HHV (MJ/kg) = −0.03ASH—0.11 M + 0.33VM + 0.35FC. This correlation has considered the dependence of calorific value on fixed carbon, volatile matter, ash, and moisture.

The calorific value at 30 minutes from the predictive equation is 30% higher compared with the result in this study for all the temperatures considered. Probably at 30 minutes, in the process of heating, some raw materials (not burn perfectly) still remain, which is indicated by the calorific value still far below the value obtained from the theoretical calculation. Heating time of 60 minutes shows the difference between predictive formulas and those in the present study to be about 40% for temperatures 250°C and 300°C. Temperature 350°C shows the differences decrease from about 30% for 30 minutes to 15% for 60 minutes and become similar for 90 minutes, which may be due to the higher ash content, which indicated that the predictive calorific value from Majumder et al.'s study [27] was valid for highest ash content of bamboo leaf biomass.

Table 3 shows elemental composition from EDS for carbon and oxygen, and Table 4 shows the details of all the elements detected by EDS. We added the result from the predictive equation in Table 3 for elemental composition (carbon, hydrogen, and oxygen) by using binary or higher than binary correlations proposed by Parikh et al., Shen et al., and Nhuchhen et al. from proximate analysis [27–30] data in Table 4 as input parameters. Parikh et al. [28] used 200 data points and validated 50 data points to find a general correlation for determining elemental composition from the simple proximate analysis. Parikh et al. [28] found a binary correlation to determine the elemental composition given as follows: C = 0.637FC + 0.455VM, H = 0.052FC + 0.062VM, and O = 0.304FC + 0.476VM with average absolute error for carbon, hydrogen, and oxygen being <5%. The limiting factor for this correlation is that it requires the sample to be in the free from ash form, meaning a preprocessing sample is needed, which is not suitable for bamboo leaf biomass at high temperature.

Shen et al. [29] used 66 data from a different category of biomass for validating 20 data points for derivation correlations to calculate the elemental composition from the simple proximate analysis with an average absolute error for carbon, hydrogen, and oxygen being <4.5%. The regression analysis model *X* = aFC + bVM + cASH was used in [29] to correlate the data, where *X* represents C, H, and, O, respectively. The least square method was used to evaluate the adjustable parameters *a*, *b*, and *c* for the prediction. They found that fixed carbon and volatile matter content affect the prediction value of C, H, and O content of biomass significantly. Ash content was also affected but it was weaker than fixed carbon and volatile matter content. Shen et al. [29] claim the accuracy of ternary correlations in their model is better than that of binary correlations by Parikh et al. [28] when predicting C and H content of biomass especially for materials with higher ash contents. The correlation of the elemental composition with data resulting from proximate analysis based on Shen et al. [29] is as follows: C = 0.635FC + 0.064VM—0.095ASH, H = 0.059FC + 0.060VM + 0.010ASH, and O = 0.340FC + 0.469VM—0.023ASH. New correlation by used data from torrefied biomass with an additional constant for determining C, H, and O is −35.9972, 55.3678, and 223.6805, respectively, proposed by Nhuchhen [30]. This correlation used 60 data, especially those biomasses which have negligible nitrogen and sulfur contents for validating with average absolute error <3% which is given as follows: C = − 35.9972 + 0.7698VM + 1.3269FC + 0.3250ASH, H = 55.3678–0.4830VM—0.5319FC – 0.5600ASH, O = 223.6805  – 1.7226VM – 2.2296FC  – 2.2463ASH. Nhuchhen [30] is suitable for analyzing the data including nitrogen at 350°C for 90 minutes time of heating but not for EDS as shown in Table 4.

From the existing correlation, we determined C, H, and O contents as shown in Table 3 and we compared them with data from an experiment using EDS for C and O. All prediction systems showed the same trend in predicting the composition of the element. As shown in Table 3, the prediction from Shen et al. [29] for carbon composition shows the lowest value compared with that of Parikh et al.'s [28] and Nhuchhen's results [30] with a difference of >20%. As can be seen in Table 3, for oxygen, it shows the same composition for temperature 250°C and 300°C but not for 350°C, although Shen et al. [29] used ternary, Parikh et al. [28] used binary, and Nhuchhen [30] used four correlations. Compositions of hydrogen are similar for heating time 60 minutes for all temperatures considered in this study. Carbon, silicon, and nitrogen contents determined by EDS in this study show the time of heating is an important parameter which shows the carbon and nitrogen contents were decreasing with increases in the heating time but not for oxygen. The amount of oxygen from EDS is highly different compared with the oxygen from the predictive equation for about 30–70% from low to high temperature. The carbon content shows good agreement between experiment data and EDS data with the predictive equation from Parikh et al.'s [28] and Nhuchhen's [30] data. These indicated the existing correlation from Parikh et al. [28] and Nhuchhen [30] shows high accuracy for predicting carbon composition from bamboo leaves. As can be seen in Table 4, the main composition for biochar from bamboo leaves is C, Si, and O. The temperature and time of heating resulting in highest ratio of C/Si about 2.56 are 300°C and 30 minutes, respectively, which is the best parameter for low-cost carbon/silicon material. For silicon (Si), the content is increased with increasing temperature and time of heating because the melting point is highest compared with another element. The production of high content Si from bamboo leaves should use highest temperature (below melting temperature) and time of heating.  Table 5 shows the carbon composition from coconut shell, mango wood [31], rice husk [32], corn stoves [33], coconut fiber, and paddy straw [34] for comparison. Carbon composition of bamboo leaves at 300°C and 30 minutes of heating shows higher carbon composition compared with biomass from rice husk [32] and paddy straw [34].

The important characteristics required in the process of energy production are the amount of volatile matter and ash content should be low as possible. The high carbon and calorific values can increase the energy production, and thus, biochar in this study for 300°C and 30 minutes becomes attractive as an energy source. Small amounts of N will reduce NO_x_ emissions as a source of pollution and corrosion [17]. The high amount of ash, in this study for ≥60 minutes and 350°C of heating, is affected by the reduction of energy production and an increase of handling cost for the disposal process. Moisture content is high which will aggravate the chemical properties of the fuel, hence complicating the process of energy production [17]. In this study, the higher energy prediction for bamboo leaves based on carbon content, calorific value, and ash is at 30 minutes and 300°C of heating.

**Figure 1:**
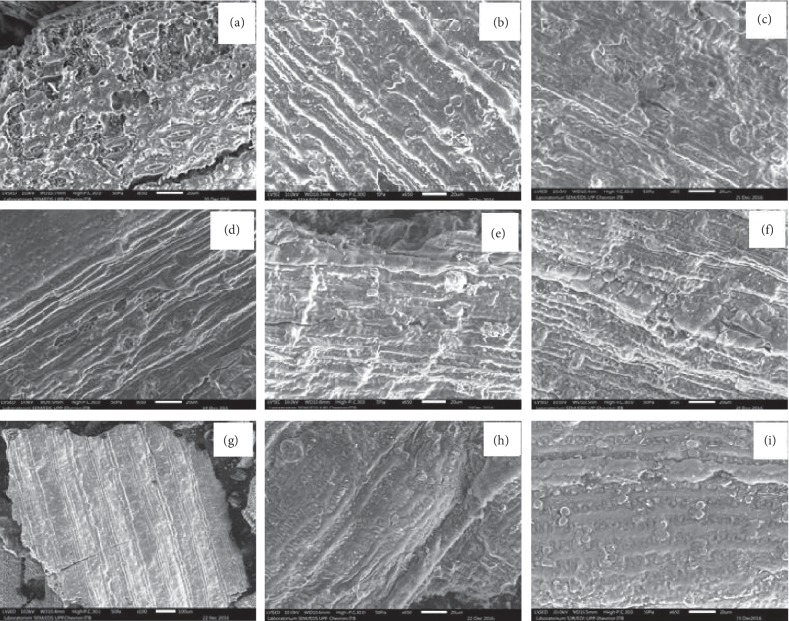
Scanning electron microscopy (SEM) image as a function of temperature and time of heating in oxygen-free environments. 250°C, 300°C, and 350°C for (a, d, and g) 30 minutes, (b, e, and h) 60 minutes, and (c, f, and i) 90 minutes.

Figure 1 shows the SEM image as a function of temperature and time of heating in oxygen-free environments. 250°C, 300°C, and 350°C for (a, d, and g) 30 minutes, (b, e, and h) 60 minutes, and (c, f, and i) 90 minutes. The characteristics of surface morphology are sheets that may contain carbon in the form of graphene.

**Figure 2:**
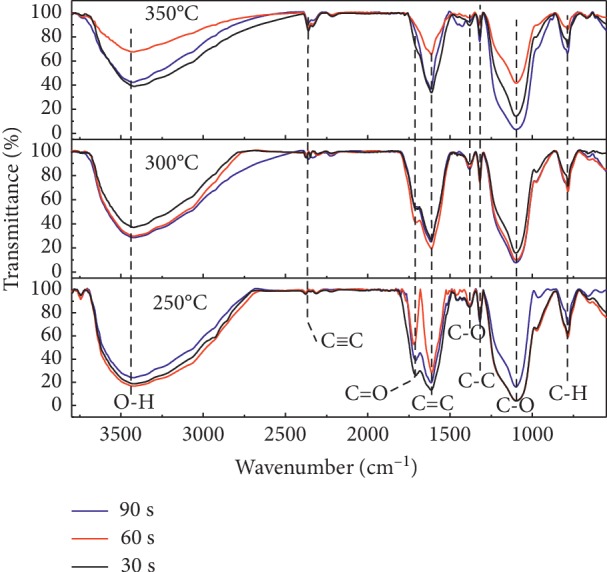
Fourier-transform infrared spectra (FTIR) spectra for biochar from bamboo leaves as a function of wavenumber for different temperatures and times of heating (see Table 6 for the detailed chemical bonding and intensity detected by FTIR).

Fourier-transform infrared (FTIR) spectroscopy was used to analyze the effect of temperature and time of heating to the surface functional groups of biochar. Besides porosity, the adsorption behavior is influenced by the chemical reactivity of the surface especially in the form of chemisorbed oxygen in various forms of functional groups. The temperature effect on biochar was successfully investigated by using FTIR but not a significant change in intensity with time variation.  Figure 2 shows the FTIR spectra as a function of temperature and time of heating in oxygen-free environments. More clear information about chemical bonding, peak position, and intensity from Figure 2 is shown in Table 6. Most of the FTIR gives features commonly from organic functional groups, which are used for examining the organic components of biochar. The peak at 3430 cm^−1^ would be expected from organic O-H stretching with contribution from any water molecule that may remain in the sample or other minerals derived from the hydroxyl group. The decrease in intensity of the hydroxyl peak for the temperature increase from 250°C to 350°C indicates that loss of hydrogen and oxygen atoms due to the breaking bond from the hydroxyl group. The band located at 2361 cm^−1^ is related to the C≡C stretching of alkynes in hemicellulose being pronounced clearly at the highest temperature. The aromatic group from lignin gives rise to C=C asymmetric stretching at 1614 cm^−1^ which indicated a *G* band in [6], [16], and [23] corresponding to the sp2-hybridization bonding of carbon atoms. C-H bending modes at 856 cm^−1^ decrease and produce CH_4_ as a gas with the temperature increasing from 250°C to 350°C [16, 23, 35].

As can be seen in Figure 2 and Table 6, the vibration of C=O stretching of a cyclic and acid anhydride is ascribed high intensity at 1670 cm^−1^ for temperature 250°C, as the temperature increases the intensity decreases and disappear at 350°C due to thermal degradation with substantial loss of oxygen atoms and produced CO_2_ gas. This is consistent with the result of hydrogen and oxygen content from the predictive equation and EDS data in Table 3. As the heating temperature increases to 300°C, the breaking bond of C=O may be also increased and produce C≡C bond as a result. The transmittance at 1383 cm^−1^ is due to the sp3-hybridization bonding of carbon atoms as discussed by Chen et al. [6], Siengchum et al. [16], Okolo et al. [22], Chee et al. [23], and Grover et al. [34], and transmittance at 1098 cm^−1^ is due to symmetric C-O stretching for cellulose, hemicellulose, and lignin. The transmission peak of 1319 cm^−1^ recommends the occurrence of aromatic with C-C stretching (ester and phenol) [34, 35]. On the other hand, the peak observed at 784 cm^−1^ reveals alkynes with C-H bending being present. The FTIR was confirmed as all cellulose, hemicellulose, and some lignin content in bamboo leaf biomass-assigned peaks increase with increase in the heating temperature producing CO_2_ and CH_4_.

**Figure 3:**
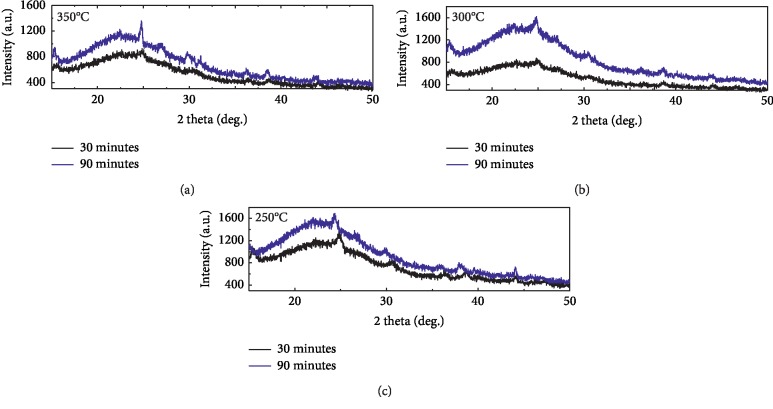
X-ray diffraction (XRD) patterns of the biochar from bamboo leaves for heating times 30 and 90 minutes with temperatures 250°C, 300°C, and 350°C.

Figure 3 shows the XRD patterns of the biochar for heating times 30 and 90 minutes with temperatures 250°C, 300°C, and 350°C. They are typical of disordered carbons which shows high carbon content bonding with silicon as can be seen clearly by the broad (002) reflections between 20° and 30°. The characteristic of 100 peaks at 44° assigned as graphite is still discernible at lowest temperature which indicates small domains of coherent and parallel stacking of the graphene sheets. According to [4, 22], the empirical parameter, *R*, is defined as the ratio of the height of the (002) Bragg reflection to that of background and is a measure of the quantity of single-layered carbon sheets in disordered carbons, which is consistent with surface morphology in Figure 1 from SEM image. The values of *R* are decreased with increase in the temperature and time of heating. High values of the *R* factor for low temperature suggest large concentrations of nonparallel single layers of carbon. The increase in the *R* value at high temperature and time of heating suggests a breakdown of aligned structural domains in the carbon matrix.

**Figure 4:**
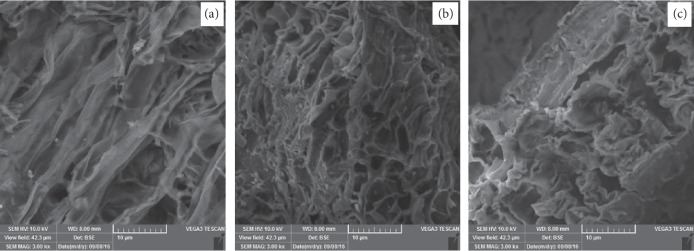
Scanning electron microscopy (SEM) images of activated biochar from bamboo leaves in oxygen-free environments at 30 minutes heating for temperatures (a) 250°C, (b) 300°C, and (c) 350°C.

Figure 4 shows the SEM image of activated biochar from bamboo leaves by using KOH as activating agent for 24 hours and cleaning with distilled water until neutral pH in oxygen-free environments at (a) 250°C, (b) 300°C, and (c) 350°C with 30 minutes of heating. We can see clearly from the figure that the temperatures 300°C and 350°C show a big amount and a small amount of micropore form, respectively. Temperature 250°C shows laminar (tube) structure without pore. This result shows 30 minutes and 300°C of heating result in the big amount of micropore with highest ratio of C/Si as shown in Table 4, which indicated the potential for energy application consistent with the calorific value. Based on Ref [36] was reported that the surface structure was influenced to the porosity and the functional group on the surface which may contribute to the formation of graphite structure and elemental composition.

## 4. Conclusion

Chemical and physical properties of bamboo leaves were analyzed by means, proximate analysis, SEM-EDS, FTIR, and XRD. Proximate analysis shows that ash composition for temperatures 300°C and 350°C increases with increasing heating time due to the release of CO_2_ and CH_4_ gases coming from hemicelluloses, cellulose, and lignin decomposition. EDS data for carbon show the differences about 15% at low temperature to 10% at high temperatures with the predictive equation data, which indicated that the existing correlation shows better accuracy in predicting carbon composition in this study. Carbon and nitrogen content determined by EDS shows the time of heating is an important parameter which shows the carbon and nitrogen contents were decreasing with increase in the heating time, and oxygen content increased with an increase in the heating time. XRD shows broad (002) reflections between 20° and 30°, and surface morphology from SEM indicated disordered carbon with small domains of coherent and parallel stacking of the graphene sheets. FTIR shows that C≡C stretching of alkynes pronounced clearly at the highest temperature, and the aromatic group corresponds to the sp2-hybridization bonding of carbon atoms C=C asymmetric stretching at 1614 cm^−1^. This means that the characteristic of chemical composition, calorific value, bonding formation, amount of micropore, and structural properties of the bamboo leaves for 30 minutes and 300°C of heating show to be the best parameters for low-cost carbon material and prominent source of useful energy.

## Figures and Tables

**Table 1:** Percentage of volatile matter, moisture, ash, and fixed carbon of bamboo leaves with heating temperatures and times of 250°C, 300°C, and 350°C for 30, 60, and 90 minutes, respectively.

Temperature (°C)	Time (s)	Moisture content (%)	Volatile matter (%)	Ash content (%)	Fixed carbon (%)
250	30	2.72	32.21	34.15	30.57
	60	2.88	30.46	41.66	24.81
	90	4.40	38.22	32.27	24.63

300	30	6.63	29.71	36.64	27.35
	60	2.31	31.22	42.27	24.45
	90	3.50	28.33	45.70	22.26

350	30	2.40	25.94	50.10	21.40
	60	1.96	28.59	60.43	9.17
	90	2.87	22.32	71.37	3.89

**Table 2:** Calorific value determined by using the bomb calorimeter in this study compared with that of the predictive model developed by Majumder et al. [27].

Temperature (°C)	Time (s)	Calorific value (MJ/Kg)	Reference [27]
250	30	14.82	19.91
	60	9.21	17.16
	90	5.74	20.11

300	30	10.85	17.37
	60	7.68	16.96
	90	6.67	15.23

350	30	7.94	14.26
	60	7.03	10.69
	90	5.80	6.52

**Table 3:** Elemental composition determined by energy-dispersive spectroscopy (EDS) for carbon and oxygen (see Table 4 for the details of all elements detected by EDS).

Element	Temperature (°C)	Time (s)	Reference [30]	Reference [29]	Reference [28]	Experimental
Carbon	250	30	41.21	18.16	35.13	29.68
		60	35.13	13.34	29.06	30.87
		90	36.95	14.53	35.76	28.50
	300	30	34.22	15.44	30.59	41.49
		60	36.04	13.62	29.34	30.59
		90	30.59	11.81	27.52	27.52
	350	30	30.10	10.39	25.07	30.01
		60	18.37	3.05	20.20	20.20
		90	9.19	-3.01	12.21	11.66

Hydrogen	250	30	4.18	4.06	3.61	—
		60	3.99	3.67	3.10	—
		90	5.52	4.11	3.67	—
	300	30	6.12	3.64	3.25	—
		60	3.76	3.76	3.38	—
		90	4.14	3.51	2.94	—
	350	30	3.33	3.39	2.69	—
		60	2.62	2.88	2.30	—
		90	2.75	2.24	1.54	—

Oxygen	250	30	21.59	25.44	24.88	34.22
		60	22.73	22.16	22.16	40.22
		90	29.80	26.01	25.44	42.43
	300	30	29.49	21.78	22.92	34.41
		60	20.14	22.35	21.78	36.56
		90	21.78	20.14	20.71	40.98
	350	30	18.56	17.49	18.06	38.83
		60	16.92	15.28	16.35	39.40
		90	16.35	9.28	10.92	47.61

**Table 4:** The chemical composition of bamboo leaves as a function of temperature and time of heating was measured by using energy-dispersive spectroscopy (EDS).

Element (%)	250°C/time (min)			300°C/time (min.)			350°C/time (min.)		
	30	60	90	30	60	90	30	60	90
C	31.38	31.2	28.93	41.5	30.32	27.92	30.31	21.36	11.5
Si	28.13	24.43	26.34	16.19	27.82	25.96	24.43	33.15	35.78
Ratio C/Si	1.12	1.28	1.09	2.56	1.09	1.08	1.24	0.64	0.32
O	34.2	40.41	41.56	34.45	35.8	40.45	38.58	39.88	47.36
N	2.03	1.87	1.47	2.72	1.42	1.02	2.1	0.59	
Na					0.01		0.02		0.16
Mg	0.43	0.29	0.07	0.27	0.55	0.71	0.24	0.7	0.94
Al	0.84	0.34	0.28	0.65	2.76	1.01	1.71	1.24	1.06
K	2.71	1.17	0.44	2.86	1.73	2.08	2.03	2.52	2.59
Ca	0.28	0.28	0.91	0.36		0.44	0.59	0.54	0.63

**Table 5:** Carbon composition from different references determined by ultimate analysis for comparison and bamboo leaves in the present study (PS) as a function of temperature and time of heating measured by energy-dispersive spectroscopy (EDS).

Raw materials	C (%)	Ref.
Coconut shell	50.22	Channiwala, 1992 [31]
Rice husk	34.60	Lanh et al., 2016 [32]
Mango wood	46.24	Channiwala, 1992 [31]
Corn stover	46.50	Gregory et al., 1982 [33]
Coconut fiber	46.43	Grover et al., 2002 [34]
Paddy straw	35.97	Grover et al., 2002 [34]
Bamboo leaves	PS (present study)	
250°C, 30 minutes	31.38	PS
300°C, 30 minutes	41.50	PS
350°C, 30 minutes	30.31	PS

**Table 6:** Peak position and intensity of chemical bond for bamboo leaves as a function of temperature and time of heating were measured by using Fourier-transform infrared (FTIR).

Chemical bond	250°C peak position and intensity (time (minutes))	300°C peak position and intensity (time (minutes))	350°C peak position and intensity (time)
O-H stretching (lignin, hemicellulose, and cellulose)	3420 cm^−1^, 18.65 (30), 16.67 (60), 23.95 (90)	3420 cm^−1^, 37.03 (30), 29.77 (60), 28.44 (90)	3420 cm^−1^ 38.98 (30), 67.58 (60), 42.28 (90)

C≡C asymmetric stretching (hemicellulose)	2375 cm^−1^ 96.10 (30), 97.70 (60), 97.17 (90)	2375 cm^−1^ 94.65 (30), 97.03 (60), 92.86 (90)	2360 cm^−1^ 84.88 (30), 86.90 (60), 91.18 (90)

C=O stretching (lignin)	1710 cm^−1^ 25.82 (30), 52.71 (60), 38.59 (90)	1705 cm^−1^ 52.01 (30), 41.23 (60), 53.82 (90)	Peak not observed

C=C asymmetric stretching (lignin)	1614 cm^−1^ 13.20 (30), 28.37 (60), 19.62 (90)	1614 cm^−1^ 26.94 (30), 41.23 (60), 53.82 (90)	1614 cm^−1^ 33.71 (30), 65.49 (60), 36.82 (90)

C-O stretching (hemicellulose)	1383 cm^−1^ 85.03 (30), 87.05 (60), 85.93 (90)	1382 cm^−1^ 89.24 (30), 86.93 (60), 84.94 (90)	1382 cm^−1^ 89.41 (30), 95.88 (60), 92.62 (90)

C-C stretching (hemicellulose)	1319 cm^−1^ 74.09 (30), 75.32 (60), 72.41 (90)	1318 cm^−1^ 75.56 (30), 74.20 (60), 78.37 (90)	1319 cm^−1^ 80.92 (30), 89.61 (60), 76.14 (90)

C-O stretching C-OH bending (hemicellulose, cellulose)	1097 cm^−1^ 3.87 (30), 4.24 (60), 16.08 (90)	1098 cm^−1^ 15.76 (30), 9.95 (60), 7.76 (90)	1098 cm^−1^ 14.21 (30), 41.57 (60), 3.21 (90)

C-H bending (lignin)	784 cm^−1^ 62.18 (30), 59.01 (60), 69.85 (90)	784 cm^−1^ 71.25 (30), 66.72 (60), 67.82 (90)	785 cm^−1^ 73.56 (30), 86.10 (60), 66.46 (90)

## Data Availability

The data used to support the findings of this study are available from the corresponding author upon request.
